# Using Typhoid Conjugate Vaccines to Prevent Disease, Promote Health Equity, and Counter Drug-Resistant Typhoid Fever

**DOI:** 10.1093/ofid/ofad022

**Published:** 2023-06-02

**Authors:** Nginache Nampota-Nkomba, Megan E Carey, Leslie P Jamka, Natalie Fecteau, Kathleen M Neuzil

**Affiliations:** Blantyre Malaria Project, Kamuzu University of Health Sciences, Blantyre, Malawi; Cambridge Institute of Therapeutic Immunology and Infectious Disease, Department of Medicine, University of Cambridge, Cambridge, UK; Center for Vaccine Development and Global Health, University of Maryland School of Medicine, Baltimore, Maryland, USA; Center for Vaccine Development and Global Health, University of Maryland School of Medicine, Baltimore, Maryland, USA; Center for Vaccine Development and Global Health, University of Maryland School of Medicine, Baltimore, Maryland, USA

**Keywords:** drug-resistant *Salmonella* Typhi, health equity, typhoid conjugate vaccines, typhoid fever

## Abstract

Typhoid fever is a serious disease that disproportionately impacts children in low-resource settings in sub-Saharan Africa, South and Southeast Asia, and the Western Pacific. The prevalence of antimicrobial-resistant strains of *S.* Typhi continue to increase worldwide. Two safe, effective, and cost-effective typhoid conjugate vaccines (TCVs) are World Health Organization-prequalified for the prevention of typhoid fever in children as young as 6 months. Typhoid conjugate vaccines have proven effectiveness in preventing drug-resistant *S.* Typhi and have been deployed successfully in outbreak response and routine immunization scenarios. Broad and equitable distribution of TCVs is essential to combat the spread and potentially devastating consequences of typhoid fever. It is vital to empower decision-makers in typhoid-endemic countries to introduce TCVs and for leaders to embrace this critical tool to prevent typhoid fever, slow the spread of drug-resistant *S.* Typhi strains, promote health equity, and save lives.

## Teaser

Typhoid conjugate vaccines (TCVs) are safe, effective, and WHO-prequalified for children as young as 6 months. TCVs have the potential to prevent typhoid fever and advance health equity, while simultaneously slowing the emergence and spread of drug-resistant *Salmonella* Typhi strains.

For much of the world, typhoid fever—a serious enteric fever caused by *Salmonella enterica* serovar Typhi (*S.* Typhi)—is a distant memory. Before the 1940s, typhoid fever was prevalent in major cities across the globe but became a “disease of the past” after improvements in water quality and sanitation, and the advent of antibiotics [1, 2]. Unfortunately, typhoid fever remains a threat in much of the world, disproportionately affecting children from low-resource areas of sub-Saharan Africa, South and Southeast Asia, and the Western Pacific, where, despite decades of investments, water and sanitation infrastructure are insufficient and contaminated food and water are prevalent. The 2019 Global Burden of Disease Study estimates that *S.* Typhi caused more than 9 million cases and more than 110 000 deaths globally, with the majority of severe disease occurring in children in low-resource settings in Asia and sub-Saharan Africa [3]. Increasing urbanization and climate change raise concerns that these numbers may continue to rise. Although typhoid is treatable with antibiotics, resistance to all oral antimicrobials licensed to treat typhoid fever has been reported, making typhoid prevention and control a pressing global issue [4, 5].

Noting the continued high burden of typhoid fever and the alarming increase in the prevalence of antimicrobial resistance (AMR) in low- and middle-income countries (LMICs), in 2018, the World Health Organization (WHO) released updated recommendations for the use of typhoid vaccines to prevent typhoid fever [6]. The WHO recommends a single dose of typhoid conjugate vaccine (TCV), as part of routine immunization, in typhoid-endemic countries for children 6 months of age and older, plus catch-up vaccination for children up to 15 years of age where feasible. The WHO recommendation specifies that the age of TCV administration, target population, and delivery strategy for routine and catch-up vaccination should be based on the local epidemiology, including AMR patterns, and programmatic considerations of a country's routine childhood immunization program. The WHO also recommends prioritization in countries with the highest burden of disease or a high burden of drug-resistant *S.* Typhi. More importantly, Gavi, the Vaccine Alliance (Gavi) opened a funding window for TCVs and committed US $85 million of support for TCV introduction in Gavi-eligible countries [6].

**Figure 1.**
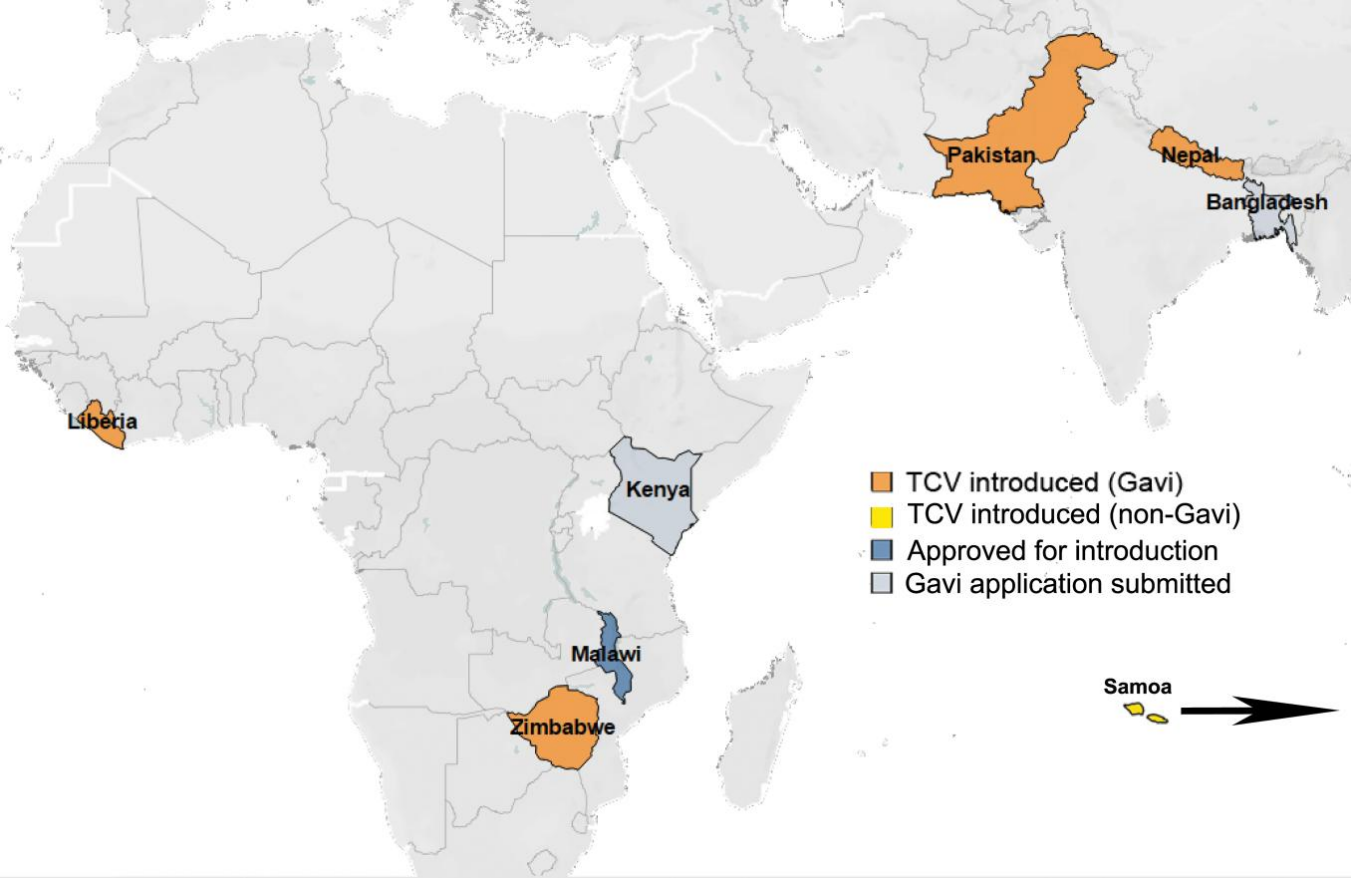
Countries that have introduced typhoid conjugate vaccines (TCV), are approved for introduction, and have submitted an application to Gavi as of September 2022.

The WHO reiterated their support for existing TCV recommendations in 2022 after review of updated data from population-based surveillance studies, clinical trials, and early country introductions [7]. Data from clinical studies among children in diverse epidemiologic settings in Malawi [8], Nepal [9], Bangladesh [10], and India [11] show TCVs are safe, well tolerated, and approximately 80% efficacious up to 3 years after a single dose [12]. Furthermore, efficacy has been demonstrated in all pediatric age groups, including children younger than 2 years of age [10]. Importantly, data from Malawi and Burkina Faso [13–15] support TCV coadministration with routine childhood vaccines, including measles-rubella and yellow fever vaccines at 9 months and group A meningococcal conjugate vaccine and measles-rubella vaccine at 15 months of age. Likewise, studies in Asia report no altered immune response when TCV was coadministered with measles-containing vaccines [16]. This pool of information informed TCV country introductions in Pakistan, Liberia, Zimbabwe, and Nepal, planned introduction in Malawi, and decisions to submit Gavi applications in Bangladesh and Kenya (Figure 1). Postvaccine introduction evaluations under real-world conditions in Pakistan and Zimbabwe demonstrate high effectiveness against extensively drug-resistant (XDR) strains [17] and in outbreak situations, respectively [18–20]. Study populations under follow up will continue to inform our understanding of the durability of antibody responses [15, 21] and vaccine effectiveness [8].

Although the battle against typhoid is an ancient one [22], we currently face a confluence of factors that elevate the urgency for better prevention and control. These challenges include the increased prevalence and severity of AMR, climate change, the coronavirus disease 2019 (COVID-19) pandemic with resultant disruptions to primary healthcare access and immunization services as well as widespread misuse of antimicrobial agents to treat COVID-19 in some settings, and an increase in health disparities. In this article, we discuss the potential of TCVs to prevent typhoid fever and advance health equity, while simultaneously slowing the emergence and spread of drug-resistant *S.* Typhi strains. In addition, we make the case for developing new, low-cost diagnostic tools to support decision making around TCV introduction in settings where blood culture is not widely available. Now more than ever, with climate change, rapid urbanization, and population displacement potentially leading to higher typhoid transmission, and increasing prevalence of AMR threatening effective outpatient treatment, it is imperative that we arm decision makers in typhoid-endemic countries with the tools needed to deploy preventative, life-saving interventions like TCVs.

## ANTIMICROBIAL RESISTANCE

Antimicrobial resistance is one of the most important modern threats to global public health. A 2019 systematic analysis of the global burden of bacterial AMR estimated 4.95 million deaths associated with bacterial AMR and 1.27 million deaths attributable to bacterial AMR [23]. The all-age mortality rate attributable to infections caused by drug-resistant pathogens was highest in sub-Saharan Africa, particularly in Western sub-Saharan Africa. Of the 23 pathogens evaluated, *S.* Typhi was ranked 11th highest, with 23 700 deaths attributable to infections caused by drug-resistant strains.

Antimicrobial resistance has posed a challenge to effective typhoid control since the advent of antimicrobial therapy in typhoid treatment, beginning with chloramphenicol resistance [24]. Multidrug-resistance ([MDR] resistance to first-line antimicrobials chloramphenicol, trimethoprim-sulfamethoxazole, and ampicillin) emerged in the 1960s and became widespread in Asia by the late 1980s [4]. Fluoroquinolones were then used as first-line treatment of typhoid in the region, leading to the emergence of decreased susceptibility to fluoroquinolones, which became particularly common in South and Southeast Asia [4, 25]. Resistance to third-generation cephalosporins has subsequently been reported in Asia, including the emergence of a new XDR strain (MDR and fully resistant to fluoroquinolones and third-generation cephalosporins) in Pakistan [17, 26], leaving azithromycin as the only remaining effective oral antimicrobial against this strain. Azithromycin resistance was subsequently identified in Bangladesh [5] and reported in Pakistan, India, and Nepal [27–29]. Thus, the prospect of untreatable typhoid looms large in South Asia, where resistance to each of these antimicrobials has been reported, but the threat is not restricted to this region. Extensively drug-resistant typhoid belongs to the globally successful H58 lineage, which has spread previously to East and sub-Saharan Africa [30], further elevating the urgency for typhoid prevention and control measures in South Asia.

A recent systematic review showed that although MDR typhoid persists in Asia, it is most prevalent in Africa. The prevalence of fluoroquinolone nonsusceptible (FQNS) typhoid tends to be highest in Southeast Asia compared to other regions, although FQNS is becoming increasingly prevalent on the African continent as well [31]. Even without the presence of XDR or untreatable typhoid, the presence of drug-resistant *S.* Typhi limits treatment options. In many areas, children with MDR, FQNS, or XDR typhoid may not be able to access second-line oral therapies or may require inpatient treatment with intravenous carbapenems at a major cost, making prevention all the more important.

Both inappropriate use of antibiotics and lack of access to antibiotics are key public health problems. Lack of access, or delayed access, to antibiotics is an important contributing factor to high mortality in young children in LMICs, and increasing rates of AMR threaten to limit the positive health impact of antibiotics on reduction of child mortality. In a recent spatial modeling study, researchers analyzed household surveys and large-scale databases to estimate antimicrobial use and consumption rates in 204 countries over a 19-year period [32]. Large national and subnational variations of antibiotic usage in LMICs were noted, with the lowest levels estimated in sub-Saharan Africa and the highest in eastern Europe and central Asia. Increased consumption levels of fluoroquinolones and third-generation cephalosporins in North Africa, the Middle East, and South Asia were also identified. In addition to overuse of antimicrobials in some regions, a lack of readily available and appropriately sensitive diagnostic tools may lead to empirical diagnoses and inappropriate use of available antibiotics, which may also contribute to the emergence of AMR.

**Figure 2.**
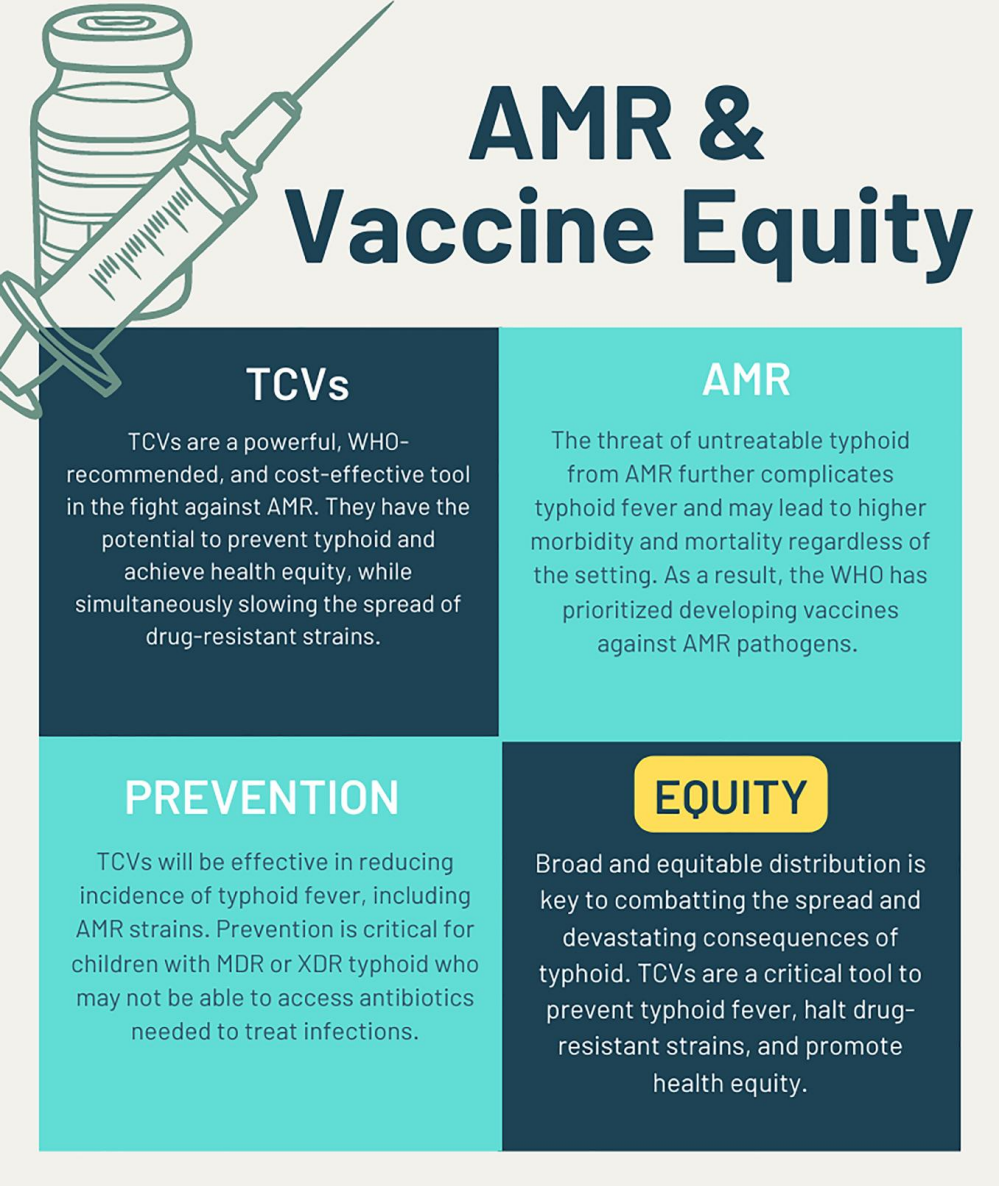
Antimicrobial resistance (AMR) and vaccine equity. MDR, multidrug resistance; TCV, typhoid conjugate vaccine; WHO, World Health Organization; XDR, extensively drug resistant.

Typhoid conjugate vaccines are a powerful, WHO-recommended, and cost-effective tool in the fight against AMR [33]. Deploying TCVs at scale would have both the direct effect of preventing drug-sensitive and drug-resistant typhoid infections, as well as the indirect effect of decreasing the overall number of antibiotics used through the prevention of some degree of syndromic illness that would be treated with antimicrobials (Figure 2). Typhoid conjugate vaccines are effective in preventing drug-resistant *S.* Typhi [33], have been used successfully in outbreak response [18] and routine immunization scenarios [8–10], and can potentially prevent further evolution of drug-resistant strains [33]. Global economic analyses predict that TCV introduction is cost-effective in many countries, including Malawi [34].

Kaufhold et al [35] developed a model to predict the impact of TCVs on AMR. Increasing coverage of TCVs decreased the total number of AMR typhoid infections, with the overall proportion of AMR cases likely remaining the same. The model was parameterized to include assumptions around relative fitness of resistance strains, the prevalence of chronic carriers, and rates of recovery without treatment [35]. An additional study combined output from mathematical models of typhoid transmission, with estimates of AMR from meta-analyses, to predict the burden of AMR prevalence of typhoid fever across 73 lower-income countries eligible for support from Gavi [36]. The effect of vaccination was predicted based on forecasts of vaccine coverage. The introduction of routine immunization with TCV at age 9 months, with a catch-up campaign up to age 15 years, was predicted to avert 46%–74% of all typhoid fever cases in 73 Gavi-eligible countries. Vaccination was predicted to reduce the relative prevalence of AMR typhoid fever by 16% (95% confidence interval, 0–49), which contrasts with predictions from Kaufhold et al [35] that TCV would have the same impact on drug-resistant and drug-susceptible strains. Overall, TCV introduction into routine immunization with a catch-up campaign was predicted to avert (1) 42.5 million cases and 506 000 deaths caused by FQNS typhoid fever and (2) 21.2 million cases and 342 000 deaths from MDR typhoid fever over 10 years after introduction [36].

Leveraging vaccines to reduce antibiotic use and reduce the burden of AMR is a WHO priority (Figure 2) [37]. Vaccinations against common pathogens, such as rotavirus and influenza, or disease syndromes for which antibiotics are frequently given, have been shown to reduce overall antibiotic use [38, 39]. In addition, vaccines may target pathogens with high prevalence of resistance that may be difficult to treat owing to AMR—pneumococcal conjugate vaccines and TCVs fall into this category. Proposed WHO initiatives for pathogens in the “very high” priority category, such as *S.* Typhi, include achieving increased coverage with licensed vaccines concurrently with improvements in water, sanitation, and hygiene (WASH) [40]. The recognition of drug-resistant typhoid fever has been a driver for early TCV introduction. Pakistan was the first country to introduce TCV into their national immunization program, fueled by the ongoing threat of XDR typhoid [41]. In this setting, TCVs were 97% effective against XDR *S.* Typhi [17]. Similarly, in response to a large drug-resistant typhoid outbreak, Zimbabwe introduced TCV in Harare and high population-density suburbs in 2019, with national introduction following in 2021 [20]. Prioritizing TCVs is important in preventing further spread of drug-resistant typhoid and additional costs to families and already-strained healthcare systems. To prevent untreatable typhoid and the spread of drug-resistant pathogens, we must act now to introduce TCVs broadly, with an initial focus on introduction in areas with high prevalence rates of AMR [40].

## ENSURING HEALTH EQUITY THROUGH BROAD DEPLOYMENT OF TYPHOID CONJUGATE VACCINE

The focus of the WHO TCV recommendations is to prevent typhoid fever during childhood. At present, TCV is recommended as a single dose during the first or second year of life. Accurately predicting the risk of typhoid throughout childhood, at this early age of vaccine administration, is problematic given the dynamic spatiotemporal variability of disease incidence, the lack of surveillance in many areas, and population movement. With climate change and increasing urbanization, typhoid fever incidence is expected to increase in the coming years, especially without the implementation of preventative interventions. Likewise, population movement will affect an individual's risk of typhoid over time. For example, in the Bangladesh cluster-randomized TCV trial, approximately 205 000 people were enrolled at baseline. Seventeen months after vaccination, there were more than 150 000 births and in-migrations, over 70 000 out-migrations, and over 35 000 people moved within the study area [10]. Although frequent catch-up campaigns are possible in clinical trials, they are less feasible in public health practice. This supports broader, rather than targeted, strategies for vaccine deployment to ensure that no child is missed.

The WHO's Immunization Agenda 2030 focuses on global equitable vaccine access, with a spotlight on more inclusive campaigns [42]. Populations in underserved communities and urban slums, or refugees and other migrants, are at increased risk of typhoid. Migration poses barriers to vaccination to include access, language, logistics, financial, and cultural differences. It is critical to slow or stop disease transmission in these migrant populations to avoid outbreaks in larger communities [43].

Nationwide TCV programs, with campaigns targeting children up to 15 years of age and introduction of TCV into a country's Expanded Programme on Immunization (EPI), provide the most equitable way to deploy TCV [15]. In Gavi-eligible countries, the full cost of a catch-up campaign to 15 years of age is covered, eliminating financial barriers with this broad approach. Such a strategy may be more problematic in countries where vaccine subsidies are not available. Through political will and partnership, Samoa became the first non-Gavi country to introduce TCV nationwide in 2021 (Figure 1). This tremendous feat included campaigns to vaccinate older high-risk populations in addition to the integration of TCV into routine immunization at 12 months of age [44].

Availability of, and access to, diagnostic services are critical for effective treatment and control [45]. Although diagnosis using blood culture is the gold standard, many LMICs lack blood culture capabilities and are unable to distinguish typhoid from other febrile illnesses. This results in undiagnosed cases, and nonspecific treatments for illnesses that may or may not be typhoid. The development and deployment of accurate, inexpensive diagnostics that can be deployed in low-resource areas is essential and will facilitate treatment guidelines and TCV introduction decisions [46]. Undiagnosed and/or untreated typhoid can have devastating consequences and complications, including typhoid intestinal perforation (TIP) [47]. In Malawi, TIP seasonality was used to estimate typhoid incidence rates, demonstrating the potential feasibility of this approach in countries lacking blood culture capabilities [48]. Typhoid intestinal perforation and other serious complications of typhoid fever require access to specialized surgical care that is not available in many low-resource settings, providing another compelling reason for the introduction of TCVs.

It is vital that all children in typhoid-endemic countries receive the benefit of TCVs, regardless of where they are born or currently live. Expanding coverage of TCVs will ensure current residence is not a barrier to receiving the vaccine [49]. Increased efforts are needed to ensure high-risk groups such as refugees, nomads, and other migrants are included in vaccination campaigns and have access to routine healthcare. It is essential to vaccinate these mobile populations *because* they are mobile. Gaps and disparities in vaccine access can have detrimental consequences at the individual, population, and global level.

## INTEGRATED DELIVERY OF HEALTH SERVICES

The WHO's Immunization Agenda 2030: A Global Strategy to Leave No One Behind aims to harness existing immunization programs while improving access to life-saving strategies with the goal of saving over 50 million lives [50]. One way to improve vaccine equity is to focus on integrated delivery of health services to minimize visits and costs [51–53]. We know that TCV is safe and effective in Africa [8] and Asia [9, 10], and it does not interfere with routine childhood vaccines [13–15]. Incorporating these policies, and expanding global vaccine coverage, will contribute to equitable vaccine access.

Multiantigen campaigns offer an efficient and cost-effective way to catch children up on missed routine EPI vaccines, for example due to COVID-19, in conjunction with introducing new vaccines. Typhoid conjugate vaccines are an appropriate choice for such campaigns, which could help reduce overall delivery costs for each individual program [54]. With the identification of a wild polio case on the African continent in 2022 [55], and measles campaign delays in 15 African countries as a result of COVID-19 [56], multiantigen campaigns are an effective way to reach children with multiple interventions in a single visit. In addition, this strategy helps to strengthen health security by preventing future outbreaks. Multiantigen campaigns can lead to increased vaccine coverage and improved access to other healthcare services, as well as provide an opportunity to catch-up on routine vaccines missed previously. In 2021 in Zimbabwe, a national TCV campaign was coupled with the delivery of human papillomavirus vaccine and inactivated poliovirus vaccine as well as distribution of Vitamin A supplements, providing protection against multiple serious diseases and boosting overall immunity in recipients [57].

Previous studies describe reasons why multiantigen campaigns may be difficult to implement, such as reservations about safety and efficacy, logistical challenges of record keeping, and the additional burden on healthcare staff [54]. Although this strategy has its challenges, vaccines have a recommended target age range for children, so integrating several vaccines in 1 campaign would involve ensuring that each child qualifies for every vaccine being administered. There are also concerns about adverse events after immunization, and, as always, robust safety surveillance and postintroduction monitoring are warranted.

For vaccination campaigns to be successful, adequate supply is essential. There are currently 2 WHO prequalified TCVs; Typbar TCV, a Vi polysaccharide conjugated to a nontoxic tetanus toxoid protein carrier, and TYPHIBEV, a Vi polysaccharide conjugated to a nontoxic variant of diphtheria toxin (CRM_197_) carrier protein. There are 2 additional TCVs licensed in India and several others in various stages of clinical development [7]. The availability of additional TCVs should increase supply security and lower costs.

## CONCLUSIONS

Typhoid fever should no longer be considered a disease of the past or a “disease of the poor,” but it should be viewed as a vaccine-preventable disease. Although improvements in WASH are critical to combat typhoid and other enteric diseases, we recognize that WASH investments may be costly, delayed, and imperfect. A single dose of TCV is safe, well tolerated, and efficacious in children as young as 9 months of age across diverse settings. Broad and equitable distribution of TCVs is key to combatting the spread—and devastating consequences—of typhoid fever, and we encourage innovation and flexibility in defining disease burden. Unfortunately, global TCV introductions have been delayed by the COVID-19 pandemic. Renewed efforts are needed to ensure that all endemic countries are empowered to introduce TCV, either alone or through integration with other health services, to prevent typhoid fever and drug-resistant infections and to promote health equity.
